# Body weight, metabolism and clock genes

**DOI:** 10.1186/1758-5996-2-53

**Published:** 2010-08-16

**Authors:** Melissa M Zanquetta, Maria Lúcia Corrêa-Giannella, Maria Beatriz Monteiro, Sandra MF Villares

**Affiliations:** 1 Laboratory of Cellular and Molecular Endocrinology (LIM/25) - University of São Paulo Medical School, São Paulo, Brazil

## Abstract

Biological rhythms are present in the lives of almost all organisms ranging from plants to more evolved creatures. These oscillations allow the anticipation of many physiological and behavioral mechanisms thus enabling coordination of rhythms in a timely manner, adaption to environmental changes and more efficient organization of the cellular processes responsible for survival of both the individual and the species. Many components of energy homeostasis exhibit circadian rhythms, which are regulated by central (suprachiasmatic nucleus) and peripheral (located in other tissues) circadian clocks. Adipocyte plays an important role in the regulation of energy homeostasis, the signaling of satiety and cellular differentiation and proliferation. Also, the adipocyte circadian clock is probably involved in the control of many of these functions. Thus, circadian clocks are implicated in the control of energy balance, feeding behavior and consequently in the regulation of body weight. In this regard, alterations in clock genes and rhythms can interfere with the complex mechanism of metabolic and hormonal anticipation, contributing to multifactorial diseases such as obesity and diabetes. The aim of this review was to define circadian clocks by describing their functioning and role in the whole body and in adipocyte metabolism, as well as their influence on body weight control and the development of obesity.

## Introduction

The prevalence of obesity is growing rapidly, affecting all ages and social classes, despite all scientific efforts to clarify its causes. Excess body weight has become one of the biggest health issues today, and is principally due to increased food availability, high caloric diets and sedentary lifestyles. Recent studies have shown the importance of new discoveries regarding the intracellular mechanisms which can trigger obesity and other metabolic disturbances.

Some studies have suggested that altered patterns of sleep/wake cycle and feeding behavior were associated to 24-hour lifestyles and changes in body weight although the mechanisms by which daily rhythms are transformed into increased adiposity remain unclear [1,2].

Circadian rhythms are biological events that constantly repeat in a 24-hour period and are generated by an endogenous mechanism. This endogenous mechanism is composed of circadian clocks, including the central clock (located in the suprachiasmatic nucleus- SCN) and peripheral clocks (located in all other cells of the organism), and is defined as the intrinsic molecular mechanisms that allow the organism to adapt to changes in its environment [3]. The clocks are synchronized or adjusted to coincide with periodical environmental events such as the day/night cycle. A well-synchronized clock guarantees that all physiological and behavioral rhythms take place in a coordinated manner over the 24-hour period [4].

Many researchers are investigating the function of the circadian clocks in circadian physiology regulation. Hence, the knowledge regarding the role of peripheral circadian clocks in glucose and lipids metabolism is starting to emerge. The adipocyte has an important role in endocrine system regulations, energy homeostasis, satiety signaling and in cell differentiation and proliferation. The circadian clock of the adipose cell is also probably involved in the control of many of these functions. It is reasonable to deduce that alterations in the peripheral circadian clock of adipose tissue can induce the onset of obesity or intensify its causes and consequences, for example, by generating modifications in adipose tissue metabolism or acting on hunger/satiety and energy balance regulation. The aim of this review was to define the circadian clocks by describing their functioning, their role in the whole body, in adipocyte metabolism, as well as their influence on body weight control and the development of obesity.

### Circadian clocks

It is well known that the life of plants, animals and humans seems to be adapted to their respective environment and that each species' survival depends on the capacity of the organism to adapt in response to periodical changes. Biological rhythms have emerged in an attempt to facilitate evolution by enabling the anticipation of many regulatory, physiological and behavioral mechanisms that improve the chances of survival of both the individual and the species. In other words, due to the physiological anticipation of the stimuli of the environment, the reactions of the organism occur at the right time of day. Alterations in the natural synchrony between cycles of day/night, activity/rest, and hormonal or feeding behavior etc. can induce modifications in this highly complex mechanism of metabolic and hormonal anticipation.

**Figure 1:**
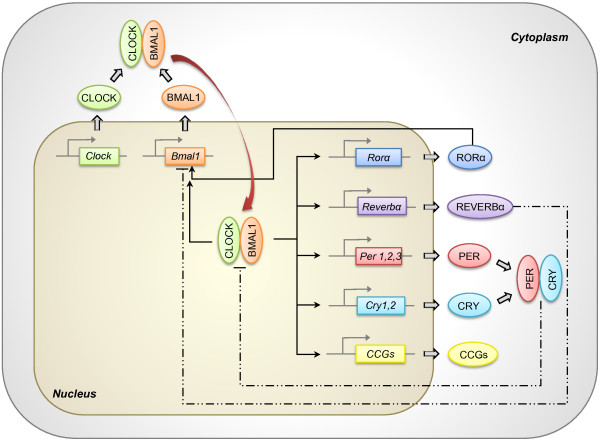
**Molecular machinery of the circadian clock**. The core clock components CLOCK and BMAL1 heterodimerize in the cytoplasm, forming a protein complex. The heterodimer is then translocated to the nucleus and binds to E-boxes on the promoter of target genes, controlling their expression. These genes include *Per1, Per2, Per3, Cry1, Cry2, Reverbα, Rorα *and many clock-controlled genes (*CCGs*). CLOCK/BMAL1 heterodimer also stimulates transcription of *Bmal1 *itself, forming the positive feedback loop of the mechanism. Negative feedback loop is mainly regulated by CRY and PER, that heterodimerize in the cytoplasm, translocate to the nucleus and inhibits CLOCK/BMAL1 transcription activity. Gene expression of *Bmal1 *is also regulated by REVERBα (inhibition) and RORα (stimulation), that compete for the same ROR elements present in the *Bmal1 *promoter. Regulation of CCGs expression by the circadian clock confers rhythmicity to a variety of molecular and physiological processes. *Straight lines: stimulation. Dashed lines: inhibition*.

The control of circadian rhythm expression involves regulation at the cellular level through the clock genes [3,5]. The clock genes codify a family of proteins that generate an auto-regulatory mechanism of positive and negative transcriptional feedback loops which occur on a 24-hour basis [3]. In mammals, the clock mechanism is composed of at least 9 main proteins: CLOCK (circadian locomotor output cycles kaput), BMAL1 (brain and muscle ARNT-like protein 1), PER1 (period 1), PER2 (period 2), PER3 (period 3), CRY1 (cryptochrome 1), CRY2 (cryptochrome 2), REVERBα (reverse erythroblastosis virus α) and RORα (retinoid-related orphan receptor- α). Many of these proteins act as transcriptional factors, since they have PAS (Per-ARNT-Sim; involved in protein-protein interactions) and bHLH (basic helix-loop-helix; involved in protein-DNA interaction) domains. With these characteristics, the proteins comprising the clock work together activating and inhibiting their own transcription. CLOCK and BMAL1 form a heterodimer that binds to E-box on the promoter of other clock genes, such as *Per1, Per2, Per3, Cry1, Cry2, Reverbα, Rorα *and many output genes that are controlled by the clock (*CCGs*: clock-controlled genes). The heterodimer CLOCK/BMAL1 also stimulates *Bmal1 *transcription, generating a positive feedback loop. Conversely, a negative feedback loop of the clock takes place through the heterodimerization of CRY and PER. The heterodimer CRY/PER translocates to the nucleus and inhibits the transcriptional activity of the CLOCK/BMAL1 heterodimer [6,7]. Nuclear receptors REVERBα and RORα participate in the regulation of *Bmal1 *expression, inhibiting or activating the transcription, respectively [8] (Figure 1).

The circadian clock components show distinct characteristics and actions on the clock's functioning. Thus, any alterations that occur in gene expression and/or protein translation may result in impaired functioning of the entire mechanism. High tissue specificity of the circadian clock also guarantees perfect working of this complex intrinsic mechanism, which is primordial for physiological and behavioral circadian responses to be triggered at the optimum time of day.

The optimization of the organism's responses at the right time of day directly depends on the synchronization between central and peripheral clocks and with regular cycles of the environment. *Zeitgebers *(from the German word, *Zeit *= time; *geber *= give) or synchronizers are the factors responsible for synchronizing the circadian clocks. Sun light determines the precise length of day and night in the 24-hour period, accurately orienting the subject in relation to the point of day. As a consequence, light is one of the most powerful synchronizers of the circadian rhythms.

Environmental light is the *zeitgeber *for the central circadian clock, being transmitted to the SCN through a neuronal pathway that starts at the retina [9]. The *zeitgebers *of the peripheral clocks are neuro-humoral factors, some of which have previously been described as glucocorticoids, and in food restriction and melatonin [9-11]. Melatonin is one of the most important neuro-humoral *zeitgebers *for the synchronization of the internal system, since it is a hormone produced and secreted only during the night by the pineal gland, which receives retino-hypothalamic neural signals that carry information about the day/night environmental cycle. Moreover, it has been suggested that social rhythms can function as *zeitgebers *for the internal system [12]. However, many critics raise questions over whether these social factors can really affect circadian rhythms independently of those controlled by environmental light.

### Peripheral clock of adipose tissue

Many studies have shown the existence of a peripheral circadian clock in adipose tissue [13,14]. Although research in this area is recent, the peripheral circadian clock of the adipose tissue seems to play a fundamental role in adipose tissue physiology and consequently in glucose and lipid homeostasis. The importance of new discoveries regarding the peripheral clock of the adipose tissue has been recognized. Some authors have recently developed an *in vitro *model for studies on circadian biology of human adipose tissue, using differentiated adipocytes derived from stem cells [15].

The adipocyte governs essential metabolic functions of the organism, not only working as an energy store but also as an endocrine organ that secrets hormones and cytokines that regulate many metabolic activities. Although circadian variations of adipose tissue metabolism have been shown to be influenced by external neuro-humoral factors [13,16], they can also be influenced internally by a peripheral circadian clock that acts on tissue metabolism and can alter adipocyte responsiveness in response to different stimuli during the day (for example, levels of glucose, insulin, fatty acids, melatonin) or can alter the capacity of lipolysis and lipogenesis [17,18].

Many adipocytokines produced in adipose tissue present circadian rhythmicity. Leptin and adiponectin show opposite patterns of circadian secretion: the peak for leptin occurs during the sleep phase of the sleep/wake cycle while adiponectin falls during the night and peaks in secretion during the morning [19-21]. In addition, it has been demonstrated that plasmatic concentrations of leptin and adiponectin also present secretion patterns of ultradian pulsatility [20,22]. Ultradian rhythms (fast repetitive oscillations within 20-hour periods) are also a significant part of the organism's temporal organization, allowing more precise adjustment of cellular and tissue responses at optimal time points for best improvement in cellular functions [23].

Adipogenesis, adipocyte differentiation and lipogenesis also appear to be directly regulated by the circadian clock. Shimba *et al*. (2005) demonstrated that adipocyte differentiation is directly regulated by BMAL1 [24], since embryo fibroblasts from *Bmal1 *knockout mice showed deficiency in the ability to become mature adipocytes, whose deficiency was restored by the transfection of an adenovirus containing the original *Bmal1*. Additionally, in the same study, when *Bmal1 *was knocked-down in cultured pre-adipocytes, the cells were unable to accumulate intracellular lipids and demonstrated decreased expression of genes related to differentiation, including the *C/ebp *family of transcriptional factors such as *Srebp1*a and *Pparγ2*, proving the importance of the circadian clock in adipocyte physiology. It was also demonstrated that *Reverbα *is a target gene for *Pparγ *[25], and that *C/ebpβ *and *Reverbα *genes are critical factors for adipocyte differentiation, showing a circadian rhythmic expression in epididymis and subcutaneous adipose tissue in mice [26].

Besides evidence showing the influence of the circadian clock on cellular proliferation and differentiation processes of adipose tissue, it has been demonstrated that hormonal and metabolic functions in adipose tissue are synchronized in circadian rhythms by the presence of melatonin in circulation [18]. Melatonin induces alterations in gene expression of the adipocyte clock genes that are translated into different cellular responses. This means that the role of the circadian clock in adipose tissue is relevant for the functioning of the tissue, modulating adipocyte intracellular responses related to external factors while also influencing overall metabolism.

### Role of circadian clocks in metabolism

It is well known that circadian clocks are involved in glucose and lipid homeostasis since many metabolic factors present circadian variations including enzymes, substrate transporters and hormones.

Evidence has demonstrated that biological rhythms and metabolism closely resemble each other. Kennaway *et al*. (2007) showed this connection [27] in a recent study on transgenic mice that presented non-rhythmical expression of clock genes in liver and skeletal muscle. The mice however, had preserved rhythmicity in the SCN and pineal gland. Although these animals did not develop obesity or fatty acid augmentation, they had an increased plasmatic adiponectin, decreased glucose transporter *Glut4 *mRNA in skeletal muscle, low glucose tolerance, low insulin levels, and a decrease in gene expression with loss of rhythmicity in enzymes related to hepatic glycolysis and gluconeogenesis.

In humans, the relationship between clock genes and metabolism has been previously demonstrated. Studies involving obese humans for instance, have found that circadian clock gene expression in adipose tissue was related to abdominal fat content and with risk factors for cardiovascular disease, since it was associated to plasmatic LDL levels, total cholesterol and with abdominal circumference [28]. The same group studied isolated adipocytes from morbid obese individuals and demonstrated that the circadian clock genes continued oscillating in a 24 hour pattern, independently of the central circadian clock, for at least 48 hours *in vitro*, and that genes directly related to adipose tissue metabolism such as PPARγ, are controlled by clock genes [29]. Moreover, it was shown that clock gene expression depends on metabolic conditions, even in healthy subjects with normal body weight [30]. It is evident that there is a direct association among rhythms at different levels of metabolism and the regulation of glucose and lipid homeostasis. We can conclude that circadian regulation is essential for maintaining metabolism balance in the organism, even though the mechanisms involved in this process are not yet well defined.

**Figure 2:**
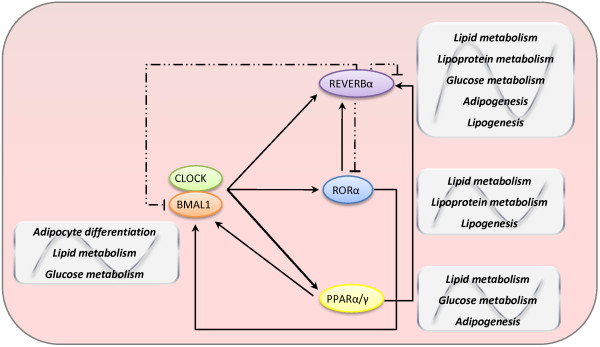
**Molecular mechanisms underlying the association between circadian clock and metabolic pathways**. CLOCK/BMAL1 heterodimer activates transcription of genes directly related to metabolism, especially nuclear receptors *Reverbα *and *Rorα *(members of clock machinery) and *Ppar *family of transcriptional factors (clock-controlled genes; do not participate of clock machinery itself). RORα and PPARα activate *Bmal1 *and *Reverbα *expressions. REVERBα represses *Bmal1*, *Rorα *and its own transcription. PPAR family and BMAL1 are involved in glucose and lipid metabolism and adipogenesis. REVERBα and RORα are implicated in the regulation of glucose, lipid and lipoprotein metabolism, adipogenesis and lipogenesis. They can also cross-talk with other important genes of metabolism such as PGC-1α (PPARγ- coactivator- 1α). *Straight lines: stimulation. Dashed lines: inhibition*.

Concerning molecular mechanisms, components of the circadian clock are linked with metabolic pathways. The gene expression of *Bmal1 *is negatively regulated by REVERBα and positively by RORα (retinoic acid receptor-related orphan receptor α), through RORE (ROR-response element) [31]. In skeletal muscle, RORα regulates lipogenesis and lipid storage [32] whereas REVERBα is stimulated during adipogenesis [33]. By contrast, the heterodimer CLOCK/BMAL1 regulates the gene expression of *Reverbα, Rorα *and *Ppar *(peroxisome proliferator-activated receptor family, principally α and γ) [34]. PPARα, which is involved in the regulation of lipid and glucose metabolism, participates in the regulation of *Bmal1 *transcription, since it is linked to the gene promoter. In addition, many other nuclear receptors directly involved in glucose and lipid metabolisms show circadian rhythmicity [35], proving the participation of different molecular mechanisms in the relationship between circadian clocks and metabolism (Figure 2).

Different biological rhythms synchronize physiological processes with daily environmental changes, allowing the organism to anticipate and adapt, generating responses in a rapid and appropriate manner. A failure in this mechanism of synchronization between rhythms and metabolism can have serious consequences, allowing or inducing metabolic pathologies such as obesity and diabetes.

Turek *et al*. (2005) have demonstrated that clock knockout mice developed obesity and showed alterations in feeding behavior such as hyperphagia, as well as hormonal abnormalities associated to metabolic syndrome, including hyperlipidemia, hyperleptinemia, hepatic steatosis, hyperglycemia and hypoinsulinemia [36]. *Bmal1 *knockout mice showed weight loss from 10 weeks of age due to a decrease in adipose and muscle tissue mass [24]. These animals also showed loss of circadian rhythm and altered metabolic phenotypes, including modified glycemic homeostasis and reduced lifetime [24,37]. Akin to *Bmal1 *knockout animals, *Per1 *knockout (*Per1*^*Brd*^) mice had reduced body mass compared to wild type animals, despite the same food intake [38]. The *Per1*^*Brd *^mice also had increased diurnal concentrations of glucocorticoids and higher fasting glucose clearance, suggesting that PER1 directly alters metabolic behavior. These results demonstrate that a disturbance in the circadian clock mechanism leads to significant alterations in glucose and lipid homeostasis and body weight maintenance.

### Circadian clocks and feeding behavior

Feeding behavior directly influences changes in body weight and is also under the circadian clocks control. Inversely, food ingestion, meals times, types of nutrient intake and metabolism can also synchronize the circadian clocks and stimulate specific responses.

Food restriction (FR) is an experimental model in which time and duration of food availability is restricted without reducing calories. Animals submitted to this regimen, being fed *ad libitum *at the same time of day for just a few hours, adjust their feeding behavior for that specific time of day within only a few days [39,40]. Thus, FR promotes physiological and behavioral modifications that show anticipatory responses from 2 to 4 hours before the feeding period. These changes are evidenced by multiple systems influenced by circadian clocks that show an increase in locomotor activity, cardiac frequency, body temperature, gastrointestinal motility and activity of digestive enzymes.

However, FR affects the peripheral circadian clocks without altering the central circadian clock, since animals without SCN submitted to FR maintain circadian rhythms independently of the light/dark cycle [41,42]. This means that FR desynchronizes peripheral and central circadian clocks yet when the feeding behavior reverts to normal, the peripheral circadian clocks are resynchronized by the central circadian clock. The hypothalamic dorsomedial nucleus, involved in central hunger and satiety regulation, is considered the food-entrainable oscillator (FEO) that acts in conjunction with the SCN to coordinate circadian feeding behavior [43].

It is known that food nutritional value also affects anticipatory responses. When rats are fed daily with two meals containing two or three macronutrients (protein + fat or protein + carb), they show anticipatory feeding behavior, a state which does not occur with free access to food containing all macronutrients [44].

Calorie restriction (CR) consists of reducing the amount of calories from lipids, carbohydrates and protein diet by 25 to 60% without causing malnutrition. In contrast, CR affects the central circadian clock, suggesting that a low calorie diet can alter physiological and behavioral rhythms. It also alters clock gene expression since it modifies the SCN function and its responsiveness to the light/dark cycle, thereby impairing circadian responses [45,46]. CR has been shown to prolong lifetime and can postpone the onset of many diseases such as obesity and diabetes [47,48].

Moreover, it was also observed that high calorie diets can influence many systems controlled by the circadian clock [49]. Animals submitted to high calorie diets develop symptoms similar to metabolic syndrome, including insulin resistance and increased body weight gain, because several hormonal and behavioral rhythms are altered by this intake [49,50]. A high fat diet also directly affects clock synchronization to environmental light carried out by the SCN [51].

The kind of food and regular feeding schedules are important synchronizers of circadian clocks since they are capable of inducing anticipatory responses and rhythms of food ingestion. The understanding of all mechanisms by which these synchronizations occur is relevant for better comprehension of the physiological processes involved in body weight and hunger/satiety regulation.

### Circadian rhythms, body weight and obesity

Generally, knowledge on body weight control is centered on the understanding of the mechanisms by which animals sense and respond to nutritional signs. In this context, circadian clocks act directly on energy balance and food intake control and consequently regulate body weight.

The season of the year influences body weight, food intake, energy consumption, body temperature among other parameters that are common in mammals as a result of evolutionary adaptations. This advantage allows animals to adapt to environment variations, optimizing their endogenous responses.

Several studies in animals have demonstrated the presence of seasonal rhythms, suggesting the importance of circadian clocks in body weight rhythmicity. Hamsters from Siberia show well-defined seasonal rhythms for body weight, food intake and reproduction. Through photoperiod modifications, i.e. length of the daylight period, seasonal alterations can be induced by melatonin secretion [52]. In the winter phenotype for instance (shorter days and longer nights) there is a decrease in food intake, lethargy, body weight loss of up to 35% (especially intra-abdominal fat loss), change to a clearer pelage and differentiated body temperature control, avoiding excessive energy expenditure [53]. There is also a decrease in leptin gene expression in adipose tissue and its concentration in circulation [54]. Studies on bird circannual rhythms have demonstrated that those exposed to shorter photoperiods exhibited lower daily energy expenditure [55]. It was also observed that, during the winter period, reindeers (arctic ruminants) exhibit lower plasmatic concentrations of leptin and insulin, together with reductions in body weight, total serum proteins and urea [56]. These, and other studies, have indicated that the photoperiod duration is associated to body weight alterations and that circadian clocks play a key role in its regulation.

It is known that light exposition at night and sleeplessness lead to an increase in adiposity and to other factors conducive to metabolic syndrome prevalence, including obesity, diabetes and cardiovascular disease.

Studies in children and young adults have shown a direct association between fewer hours of sleep and weight gain, and individuals that sleepless have an increased chance of developing obesity in adult life [57,58]. This association also holds for elderly people, and has been proven recently in a study with men and women over 65 years old [59]: individuals who slept less than 5 hours per day exhibited higher body mass index (BMI), being an average of 2.5 kg/m^2 ^higher in men, and 1.8 kg/m^2 ^greater in women.

These data were also confirmed in lab studies. One such study compared subjects submitted to 4 or 10 hours of sleep for 2 consecutive nights, and found that sleep restriction was associated with an average reduction of 18% in leptin concentration, an elevation of 28% in the orexigenic factor ghrelin, and increases in hunger (24%) and appetite (23%), especially for calorie-dense foods with high carbohydrate content [60]. Given that hunger increases during sleep restriction lead to increased food intake, individuals who sleep less are expected to experience greater weight gain over time.

Laposky *et al*. [61,62] performed studies with leptin-deficient obese *ob/ob *mice, and obese and diabetic *db/db *mice (they do not express a particular isoform of leptin receptor) and demonstrated that these animals had disturbed wake/sleep cycles, slept less and had reduced locomotor activity. Therefore, fewer sleep hours can contribute to increased prevalence of overweight and obesity through changes in regulation of appetite, increased availability of feeding and/or stimulating a decrease in energy expenditure. All these factors are influenced by the control of circadian clocks.

Night shift workers have different patterns of sleep due to changes in synchronization of their endogenous rhythms with the light/dark cycle. These sleep alterations are associated with metabolic disturbances, cardiovascular disease, diabetes and obesity. In a study involving approximately 27,500 individuals, it was observed that obesity, increased triglycerides and decreased HDL cholesterol were more prevalent in night shift workers compared to day shift workers [63]. Another study demonstrated that night workers also exhibited increased fasting glucose, free fatty acids, arterial blood pressure, abdominal circumference and BMI [64,65]. Regarding the nutritional status of night workers, different eating patterns were found: 1. total intake of calories and the presence of three major groups of nutrients in one meal did not differ from day laborers, except for a 10% higher consumption of saturated fat for night workers, 2. food intake was more fragmented during the day, being lower at breakfast and lunch, and higher at dinner and in snacks between meals, especially evening and night [65].

It is important to highlight that obese individuals report less total sleep time per night, with a difference of one hour of sleep per week corresponding to an increase of 5.4 kg/m^2 ^in BMI [1]. Sleep disorders such as insomnia and obstructive sleep apnea are very common in individuals with metabolic and endocrine pathologies, but often remain undiagnosed. This can lead to the onset of obesity and diabetes [66]. Treatment of sleep disorders has a potential to improve glucose metabolism and energy balance.

Another point to consider is that even in altered metabolic states, circadian rhythms of hormones, receptors, transporters, etc are maintained. For example, in obese subjects, circadian rhythms of leptin and adiponectin are still present, albeit changed. In a 24-hour period, obese individuals showed increased plasmatic leptin concentrations with higher peaks of secretion. On the other hand, adiponectin concentrations were lower where this was associated with smaller and shorter peaks of secretion [67]. Ghrelin (hormone involved in body weight regulation) concentrations are decreased in the obese while no increase is seen in the evening in contrast to lean subjects [67].

Recently, Kaneko *et al*. (2009) [68] demonstrated that obesity modifies clock gene circadian expression in the central nervous system. Moreover, it alters the expression of target genes of circadian clocks that can participate in mechanisms involved in the rhythmic alterations and neuronal dysfunctions observed in obese individuals such as PPARα.

Expression of peripheral clock gene components in adipose tissue are also altered in obese subjects, as demonstrated by Loboda *et al*. in 2009 [14]. The authors studied subcutaneous adipose tissue (obtained from biopsies collected in the morning, afternoon or evening) from obese patients submitted to fasting and/or treated with sibutramine. Results showed that 25% of the expressed genes in adipose tissue presented circadian rhythm, including those essentially involved in energy metabolism and tissue physiology. Thus, they hypothesized that circadian rhythms showed by the genes involved in energy metabolism may be reflecting in the adipocyte, a transition from a state of energy expenditure in the morning to a state of energy storage during the night. This mechanism was indeed demonstrated by the delay of this transition observed after fasting and treatment with sibutramine.

Therefore, obesity is closely related to circadian clock disturbances, both central and peripheral. This highlights the importance of circadian rhythms in adipose tissue physiology and energy metabolism regulation of the body. Circadian clocks play a key role in body weight control and in mechanisms implicated in this control, such as hunger/satiety cycle, feeding behavior, wake/sleep cycle, energy expenditure/storage and hormonal secretion.

### Genetics and the link between obesity, body weight and circadian clocks

Genetic factors are important contributors to obesity development, as demonstrated by several studies in humans and animals. Some authors have suggested four levels of genetic determination of obesity: genetic obesity, strong genetic predisposition, slight genetic predisposition, and genetically resistant [69].

The heritability of common obesity is usually due to an interaction of multiple candidate genes found at different locations on the gene map and is therefore polygenic in nature [70]. Candidate genes either predispose to obesity or promote body weight loss. Genes either interact with each other (gene- gene interactions) or with various environmental factors (gene- environment interactions). Interactions between biological (genes, hormones, neurotransmitters, etc.), psychobehavioral and environmental factors influence body fat accumulation and fat distribution and can also increase other elements related to body weight gain.

Most of the genes discovered to date chiefly impact hunger, satiety and food intake [71]. Mutations in the leptin gene (LEP), leptin receptor (LEPR), pro-opiomelanocortin (POMC), proconvertase 1 (PC1), melanocortin receptor 4 (MC4R), melanocortin receptor 3 (MC3R), neurotrophic tyrosine kinase receptor type 2 (NTRK2) and many others have been recognized as promoters of morbid obesity and rare forms of human obesity [72].

Studies evaluating twins and adopted children compared to their biological relatives showed heritability of BMI [73]. A recent study involving 5,092 British twins aged from 8 to 11 years revealed that genetic influences were extremely significant in BMI (77%) and abdominal circumference, whereby 60% of abdominal adiposity heritability was from the same genes in BMI, and the remaining 40% were attributed to different genetic factors [74].

Several human genetic polymorphisms have been described in circadian clock genes as have their associations with metabolic disturbances. For example, studies in different populations demonstrated some associations between polymorphisms in the *clock *gene and metabolic syndrome, hepatic steatosis, obesity predisposition, eating disorder, sleep disorders, schizophrenia and bipolar disease [75-78]. Other studies have revealed associations between: polymorphisms in BMAL1 with hypertension and type 2 diabetes [79], hypertension and NPAS2 (CLOCK gene analogous) [80], and glucose and PER2 [81].

Recent research has shown differences in the distribution of circadian clock gene polymorphism frequencies among populations in different parts of the world including Chinese, African-American, Caucasian-African individuals [82]. Population genetic analyses suggest these differences stem more from genetic factors than natural selection. These results point to the fact that each population presents variations in circadian clock genes that allow metabolic and behavioral adaptations according to their ambient, cultural and social environment.

## Conclusions

A large body of evidence suggests that obesity can cause alterations in circadian rhythms and vice-versa, since desynchronized rhythms can induce the onset of obesity and other metabolic diseases. It is clear that perfect synchronization between central and peripheral clocks and correct functioning of the adipose tissue clock are essential in regulating hunger, adiposity, energy balance and body weight. Further studies are needed to investigate the molecular and physiological mechanisms involved in this control. This knowledge can contribute to the development of new therapies for the treatment of obesity and other metabolic disorders.

## Competing interests

The authors declare that they have no competing interests.

## Authors' contributions

MMZ wrote the manuscript and performed the literature review. MLCG revised the manuscript and made suggestions for intellectual content. MBM helped in literature background and revised the manuscript. SMFV supervised the project and revised the manuscript. All authors have read and approved the final version of the manuscript.
